# Therapeutic competitions and frailty among older adults in São Paulo city: a cross-sectional population-based study, 2015

**DOI:** 10.1590/S2237-96222025v34e20240104.en

**Published:** 2025-04-11

**Authors:** Igor Gonçalves de Souza, Yeda Aparecida de Oliveira Duarte, Mariana Martins Gonzaga do Nascimento, Caroline de Godoi Rezende Costa Molino, Cristiane de Paula Rezende, Jair Lício Ferreira Santos

**Affiliations:** 1 Universidade de São Paulo, Faculdade de Saúde Pública, São Paulo, SP, Brazil; 2 Universidade Federal de Minas Gerais, Faculdade de Farmácia, Belo Horizonte, MG, Brazil; 3 University of Zurich, Centre on Aging and Mobility, Zurich, Switzerland; 4 Universidade de São Paulo, Faculdade de Medicina de Ribeirão Preto, Ribeirão Preto, SP, Brazil

**Keywords:** Aged, Frailty, Drug-Related Side Effects and Adverse Reactions, Cross-Sectional Studies, Anciano, Fragilidad, Efectos Colaterales y Reacciones Adversas Relacionados con Medicamentos, Estudios Transversales

## Abstract

**Objective:**

To identify the presence of therapeutic competitions according to the frailty component classification and to assess association between therapeutic competitions and frailty in people aged 60 or over in the city of São Paulo, Brazil.

**Methods:**

This was a population-based cross-sectional study. Frailty was identified when three to five components defined by Fried were present, and pre-frailty was identified when one or two were present. Therapeutic competitions were characterized by use of medication for a specific chronic non-communicable disease that negatively affects another disease. We used multinomial logistic regression.

**Results:**

Therapeutic competitions were identified for 13.2% of the 1,224 participants. Of this total, 18.7% were considered to be frail individuals. The most prevalent therapeutic competition involved diabetes and cardiovascular disease (4.2% of the total and 6.8% of frail people) with metformin or angiotensin-converting enzyme inhibitors. The presence of two therapeutic competitions in individuals with multimorbidity increased the likelihood of pre-frailty (odds ratio 2.51; 95% confidence interval 1.10; 5.76).

**Conclusion:**

Therapeutic competitions are common in the elderly population and more frequent among frail elderly people, with increased likelihood of pre-frailty when two therapeutic competitions are present.

## Resumen

**Objetivo:**

Identificar la presencia de competencias terapéuticas según la clasificación de componentes de la fragilidad y evaluar la asociación entre competencias terapéuticas y fragilidad en personas de 60 años o más en la ciudad de São Paulo.

**Métodos:**

Se trata de un estudio transversal de base poblacional. La fragilidad se identificó cuando estaban presentes de tres a cinco componentes definidos por Fried, y la prefragilidad se identificó cuando estaban presentes uno o dos. Las competencias terapéuticas se caracterizaron por el uso de medicamentos para una enfermedad crónica no transmisible específica que afecta negativamente a otra enfermedad. Se utilizó regresión logística multinomial.

**Resultados:**

Se identificaron competencias terapéuticas para el 13,2% de los 1.224 participantes. De esta cantidad, el 18,7% eran considerados personas frágiles. La competencia terapéutica más frecuente fue la diabetes y las enfermedades cardiovasculares (4,2% del total y 6,8% de las personas frágiles) con metformina o inhibidores de la enzima convertidora de angiotensina. La presencia de dos competencias terapéuticas en individuos con multimorbilidad aumentó la posibilidad de prefragilidad (odds ratio 2,51; intervalo de confianza del 95% 1,10; 5,76).

**Conclusión:**

Las competiciones terapéuticas son comunes en la población de edad avanzada y más frecuentes entre los ancianos frágiles, con una mayor probabilidad de prefragilidad cuando estaban presentes dos competiciones terapéuticas.

**Ethical aspects**

This research respected ethical principles, having obtained the following approval data:

Research Ethics Committee: Faculdade de Saúde Pública da Universidade de São Paulo

Opinion Number: 3,600,782

Approval Date: 26/9/2019

Certificate of Submission for Ethical Appraisal: 47683115.4.0000.5421

Informed Consent Form: Obtained from all participants before data collection.

## Introduction

The growth in the proportion of older adults in the population, in addition to the development of health technologies and the increase in life expectancy, is reflected in the increase in chronic non-communicable diseases and the growing use of medications ^1^. These changes go hand in hand with an intensification of the prevalence of frailty. Frailty is defined as a multifactorial syndrome caused by the progressive reduction of reserves and resistance to stressors, which induces functional decline and results in disabilities, dependence and greater vulnerability ^2^ ^-^ ^4^. Its consequences include increased risks to the health of these individuals, compromising independence and autonomy, in addition to increased health costs and demand for the use of more medications ^4^ ^-^ ^6^.

Although necessary in several situations, medication administered for one health condition may negatively affect another disease that is also present in the same person for which it was not directly used, thus characterizing therapeutic competition. This is characterized by three components: i) the primary disease, for which ii) the competing medication is used, and iii) the competing disease, which is negatively affected by the medication in use ^7^. Occurrence of therapeutic competition may be facilitated by the high prevalence of individuals with two or more diseases (multimorbidity) and the consequent use of medications for these conditions. Thus, the medication used for one disease may have an adverse effect on another coexisting condition. 

Prevalence of therapeutic competition has been associated with polypharmacy, as well as with poorer self-rated health, and also with occurrence of hospitalization and falls in the 12 months prior to the report given in interviews ^8^. Some of these factors, such as falls and hospitalization, are also known to increase the likelihood of frailty syndrome and even favor the process of becoming frail ^9^ ^,^ ^10^. Considering the need to expand research in the field of therapeutic competitions among the elderly population and frailty syndrome, it is relevant for clinical practice to define the factors that are associated with this phenotype. This research aimed to identify prevalence of therapeutic competitions according to the frailty component classification and to assess the association between frailty and therapeutic competitions in people aged 60 or over living in the city of São Paulo, Brazil.

## Methods

### Study design and participant

This is a cross-sectional, population-based and analytical study, carried out using data from the Health, Well-being and Aging Study (Estudo Saúde, Bem-Estar e Envelhecimento - SABE). The study population consisted of people aged 60 or over living in the city of São Paulo, of both sexes, who participated in the interviews conducted by the Study in 2015.

### Background

The SABE Study originated as a multicenter survey that, in Brazil, was conducted in the city of São Paulo and continued with multiple cohorts. It monitors the participating individuals longitudinally, whereby the structured questionnaire is repeatedly administered at regular intervals. Its objective is to assess the health status and living conditions of the elderly population, so as to assist in public health and in projecting the needs of people in this age group ^11^.

In 2000, 2,143 individuals were interviewed, composing the probabilistic sample that formed cohort A00. The individuals initially interviewed were sought each time the questionnaire was administered from then on (2006, 2010 and 2015), with the addition of a new cohort in each period comprised of individuals aged 60 to 64 years. In 2015, the cohorts resulted in 1,224 individuals interviewed. Participants were selected considering stratification by sex and age groups. The difference in the number of individuals each time the questionnaire was administered occurred as a result of deaths, failure to locate participating individuals, refusals, institutionalizations and moves to other municipalities ^11^. The complete description of the sample definition process and other methodological characteristics of the SABE Study can be consulted in the article published by Lebrão et al. (2018) ^11^.

### Dependent variable: frailty

The study dependent variable study was frailty phenotype. Its definition was based on the concept that determines the presence of the phenotype according to five components ^(3)^. In the SABE Study, these were defined as follows ^9^ ^,^ ^11^.

Unintentional weight loss - defined by the question: “In the last year, have you lost weight without going on a diet?”. A score was given if weight loss was greater than 3 kilograms.

Self-reported exhaustion or fatigue - characterized by two questions previously defined by the Center for Epidemiological Studies - Depression, and validated for Brazilian individuals ^12^: “How often, in the last week, was carrying out your routine activities a great effort in order for you to achieve them?” and “How often in the last week did you feel you couldn’t get things going?” The following results were classified: 0=never or rarely (less than one day); 1=some or little of the time (between one and two days); 2=occasionally or a moderate amount of time (between three and four days); 3=most of the time. A score was given if the answer was “2” or “3” for at least one of the questions.

Decrease in strength - determined by handgrip strength, obtained using a dynamometer. For this component, individuals forming lowest distribution quintile were considered, stratified by body mass index quartile and sex.

Reduction in gait speed - characterized by the 3-meter walk test, defined by the Short Physical Performance Battery Assessing Lower Extremity Function ^13^. Considered present in those who formed the largest distribution quintile, segmented by the median value of height and sex.

Low level of physical activity - verified by a translated version of the Physical Activity Questionnaire ^14^, by self-reporting about performance of vigorous, moderate physical activities or walking, with subsequent determination of weekly caloric expenditure on carrying out the reported activities. A score was given to those who formed the lowest quintile of caloric expenditure, stratified by sex. 

Those individuals who presented three to five frailty phenotype components were classified as “frail”. Participants with one or two components made up the “pre-frail” group. Participants were classified as “non-frail” when none of the components were present.

### Independent variables

The independent variables included those representing sociodemographic, clinical and behavioral characteristics, as follows ^9^ ^,^ ^15^ ^,^ ^16^.

Age group (in years): 60-69, 70-79, 80 or over.

Sex: male, female.

Schooling (in years): none, 1-3, 4-7, 8 or more years of study.

Living arrangement: lives with someone, lives alone.

Self-perception of income sufficiency: sufficient, insufficient.

Tobacco smoking: smoker, former smoker, never smoked.

Weekly consumption of alcoholic beverages in the last three months (times): less than 1, 1-3, 4 or more.

Health conditions: yes, no for the following chronic non-communicable diseases, obtained via disease-specific questions contained in the study questionnaire - diabetes, hypertension, depression, osteoarticular disease, osteoporosis, cardiovascular disease, lung disease, Alzheimer’s disease, hypercholesterolemia, benign prostatic hyperplasia and gastroesophageal reflux.

Medication in use: coded according to the Anatomical Therapeutic Chemical classification. Polypharmacy was defined as use of five medications or more.

Hospitalization for at least one day in the last 12 months: yes, no.

Report of at least one fall in the last 12 months: yes, no.

Self-perceived health: good/very good, regular, very poor/poor.

Presence of therapeutic competitions was used as an independent variable, previously listed by Molino (2018) through the selection of high-quality clinical practice guides listed through quality assessment and systematic review. These documents present scientific knowledge on a given subject in a summarized way, providing practical recommendations for health professionals ^8^ ^,^ ^17^ ^,^ ^18^. High-quality national and international clinical practice guides were used to identify therapeutic competitions in older adults with illnesses ^8^. In this study, therapeutic competition was verified in the group of participants with multimorbidity, present in individuals with at least two of the diseases listed above and with higher prevalence in older adults in the community ^19^.

Therapeutic competition was considered to be when the participant reported using the competing medicine and had a given primary disease in the presence of the competing disease. Other types of drug-disease interactions were not considered in this study. Fixed dose combinations were also used to identify therapeutic competitions when at least one of the drugs caused competition. 

### Statistical methods

The relative frequencies of the qualitative variables were presented in the descriptive analysis of the data, as well as the means of the quantitative variables, with standard deviations. Comparative analyses of the therapeutic competition and frailty categorical variables were carried out using Pearson’s chi-square test with Rao-Scott correction. A 5% significance level and sample weights were considered for estimates with population weightings.

Univariate and multiple analyses were performed for the group of individuals with multimorbidity, as this is the group predisposed to the presence of therapeutic competitions. Variables with a significance level less than or equal to 0.20 in the univariate analysis were selected for inclusion in the multiple model. This was carried out using multinomial logistic regression, with the frailty phenotype being the dependent variable, consisting of the “non-frail”, “pre-frail” and “frail” groups. The number of therapeutic competitions was included in the multiple model as an independent variable, categorized as “none”, “one”, “two” and “three or more therapeutic competitions”, with the “none” category being used as a reference. Statistical significance of 5% was adopted. The results were presented by means of odds ratios (OR) and 95% confidence intervals (95%CI). The Hosmer-Lemeshow quality test for multinomial logistic regression was used to assess the goodness of fit of the final multiple model, assuming the null hypothesis that the model fit is adequate ^20^. 

Stata software (version 14) was used to carry out statistical analyses. The sample weights were considered in the analyses and, therefore, the results presented can be extrapolated to the population aged 60 or over in the city of São Paulo.

## Results

In all, 1,124 individuals were included. Average age was 70.8±0.7 years, and 56.7% were female. 85.6% of participants had at least one morbidity, with hypertension (66.3%), osteoarticular disease (33.5%) and diabetes (28.2%) being the most prevalent. Of those interviewed, 28.7% reported 2 illnesses and 33.2% had 3 or more, with an average of 2.0±0.1 illnesses. Nine out of 10 individuals reported using at least 1 medication (85.4%). More than a third used polypharmacy (38.8%), and 6.4% used excessive polypharmacy (10 or more medications in use). On average, participants used 4.1±0.2 medications.

In all, 11.2% were identified as frail, 56.1% as pre-frail and 32.7% as non-frail. The majority of participants who did not use medication belonged to the pre-frail or non-frail group (97.0%). 96.2% of frail individuals and 87.7% of pre-frail individuals used at least 1 medication. Polypharmacy was found in 42.2% of individuals in this group. The highest prevalence of frail individuals was found among females, those with a lower level of schooling and those aged 80 or over (Table 1). Multimorbidity was identified in 78.9% of frail people, 62.2% of pre-frail people and 60.7% of non-frail people.

**Table 1:** Older adult characteristics (%) according to total number of interviewees and frailty phenotype. São Paulo, 2015 (n=1,224)

Variables	Total	Non-frail	Pre-frail	Frail	p-value
Sex					0.027
Female	56.7	33.5	53.2	13.3	
Male	43.3	31.7	59.7	8.6	
Age group (years)					<0.001
60-69	54.3	43.8	50.9	5.3	
70-79	30.5	25.9	64.3	9.8	
≥80	15.2	7.0	58.0	35.0	
Schooling (years)					<0.001
None	13.1	18.9	60.3	20.8	
1-3	17.9	28.4	57.6	14.0	
4-7	37.3	34.6	55.6	9.8	
≥8	31.7	38.9	54.2	6.9	
Living arrangement					0.466
Lives with someone	84.3	32.4	56.7	10.9	
Lives alone	15.7	34.9	52.3	12.8	
Perception of income					0.813
Sufficient	52.7	32.8	56.7	10.5	
Insufficient	47.3	32.8	55.5	11.7	
Health insurance					0.205
No	53.6	31.0	56.5	12.5	
Yes	46.4	34.8	55.5	9.7	
Report of at least one fall in the last 12 months					0.058
No	71.2	34.5	56.1	9.4	
Yes	28.8	28.6	55.8	15.6	
Hospitalization for at least one day in the last 12 months (n=1,220)					<0.001
No	86.8	35.1	55.8	9.1	
Yes	13.2	17.5	57.2	25.3	
Tobacco smoking (n=1.218)					0.046
Never smoked	48.3	34.2	52.5	13.3	
Former smoker	38.4	30.2	60.9	8.9	
Smoker	48.3	35.4	55.3	9.3	
Weekly consumption of alcoholic beverages in the last three months (times) (n=1,218)					0.056
<1	81.1	32.4	55.1	12.5	
1-3	11.7	37.8	56.9	5.3	
4 or more	7.2	28.3	65.4	6.3	
Self-perceived health (n=1,189)					<0.001
Very good or good	49.2	39.7	53.7	6.6	
Regular	44.1	29.4	60.1	10.5	
Poor or very poor	6.7	13.0	56.5	30.5	
Use of medications					<0.001
None	14.6	50.0	47.0	3.0	
1-2	21.9	35.4	53.8	10.8	
3-4	24.7	31.4	55.7	12.9	
5 or more	38.8	25.6	60.9	13.5	
Multimorbidity (n=1,222)					0.001
No	36.5	37.1	56.5	6.4	
Yes	63.5	30.0	55.7	14.3	
Hypertension (n=1,221)					0.009
No	33.7	37.5	54.9	7.6	
Yes	66.3	30.4	56.7	12.9	
Diabetes (n=1,221)					0.029
No	71.8	32.2	58.1	9.7	
Yes	28.2	33.6	51.5	14.9	
Lung disease (n=1,221)					0.009
No	91.8	22.5	62.5	15.0	
Yes	8.2	33.7	55.6	10.7	
Cardiovascular disease (n=1,221)					<0.001
No	76.2	36.0	54.1	9.9	
Yes	23.8	22.1	62.5	15.4	
Osteoarticular disease (n=1,221)					0.049
No	66.5	33.7	56.6	9.7	
Yes	33.5	31.5	54.3	14.2	
Depression (n=1,220)					0.025
No	84.0	33.4	56.4	10.2	
Yes	16.0	29.6	54.0	16.4	
Osteoporosis (n=1,221)					0.012
No	73.8	33.7	56.3	10.0	
Yes	26.2	27.9	54.9	17.2	
Alzheimer’s disease (n=1,220)					<0.001
No	97.9	33.4	56.3	10.3	
Yes	2.1	3.7	45.8	50.5	
Gastroesophageal reflux (n=1,222)					0.840
No	98.1	32.7	56.1	11.2	
Yes	1.9	34.2	50.8	15.0	
Benign prostatic hyperplasia (n=1,222)					0.645
No	99.8	32.7	56.1	11.2	
Yes	0.25	56.6	43.4	0	
Hypercholesterolemia (n=1,222)					0.219
No	96.6	32.3	56.3	11.4	
Yes	3.4	43.2	50.7	6.1	
Therapeutic competition (n=1.222)					0.015
No	86.8	34.5	55.0	10.5	
Yes	13.2	21.4	62.8	15.8	

We found 13.2% prevalence of therapeutic competitions among the participants, which indicates that 1 in every 10 individuals aged 60 or over in the city of São Paulo used at least 1 medication capable of negatively affecting another disease they had. Considering the total number of participants, 8.0% presented 1 therapeutic competition, 2.8% presented 2, and 2.4%, 3 or more. The percentage of individuals with therapeutic competition increased according to frailty phenotype, being 10.0% higher in frail people, compared to those who were not frail (Table 2).

**Table 2:** Older adult characteristics (%) according to presence of therapeutic competition. São Paulo, 2015 (n=1,224)

Variables	With therapeutic competition	Without therapeutic competition	p-value
Sex			0.044
Female	84.9	15.1	
Male	89.2	10.8	
**Age group (in years)**			0.058
60-69	89.2	10.8	
70-79	84.6	15.4	
80 or over	82.6	17.4	
**Schooling (years)**			0.238
None	86.6	13.4	
1-3 years	84.2	15.8	
4-7 years	85.7	14.3	
8 years or more	89.6	10.4	
**Living arrangement**			0.932
Lives with someone	86.8	13.2	
Lives alone	86.6	13.4	
**Perception of income**			0.739
Sufficient	87.1	12.9	
Insufficient	86.4	13.6	
**Health insurance**			0.499
No	87.5	12.5	
Yes	86.0	14.0	
**Report of at least one fall in the last 12 months**			0.010
No	88.7	11.3	
Yes	82.1	17.9	
**Hospitalization for at least one day in the last 12 months**			<0.001
No	88.3	11.7	
Yes	76.7	23.3	
**Tobacco smoking**			0.971
Never smoked	87.0	13.0	
Former smoker	86.5	13.5	
Smoker	86.5	13.5	
**Weekly consumption of alcoholic beverages in the last three months (times)**			0.398
<1	86.2	13.8	
1-3	88.4	11.6	
4 or more	90.9	9.1	
**Self-perceived health**			<0.001
Very good or good	91.2	8.8	
Regular	84.1	15.9	
Poor or very poor	70.4	29.6	
**Use of medications**			<0.001
None	100.0	0	
1-2	96.4	3.6	
3-4	88.3	11.7	
5 or more	75.4	24.6	
**Multimorbidity**			<0.001
No	100.0	0	
Yes	78.6	21.4	
**Hypertension**			<0.001
No	95.6	4.4	
Yes	82.3	17.7	
**Diabetes**			<0.001
No	90.8	9.2	
Yes	76.5	23.5	
**Lung disease**			<0.001
No	91.7	8.3	
Yes	32.3	67.7	
**Cardiovascular disease**			<0.001
No	92.4	7.6	
Yes	68.6	31.4	
**Osteoarticular disease**			<0.001
No	90.6	9.4	
Yes	78.7	21.3	
**Depression**			<0.001
No	88.4	11.6	
Yes	78.3	21.7	
**Osteoporosis**			<0.001
No	88.4	11.6	
Yes	78.0	22.0	
**Alzheimer’s disease**			0.956
No	86.8	13.2	
Yes	86.4	13.6	
**Gastroesophageal reflux**			0.695
No	86.7	13.3	
Yes	89.7	10.3	
**Benign prostatic hyperplasia**			0.038
No	86.9	13.1	
Yes	43.4	56.6	
**Hypercholesterolemia**			0.092
No	87.1	12.9	
Yes	78.1	21.9	
**Frailty phenotype**			0.015
Non-frail	91.4	8.6	
Pre-frail	85.2	14.8	
Frail	81.3	18.7	

**Figure 1:**
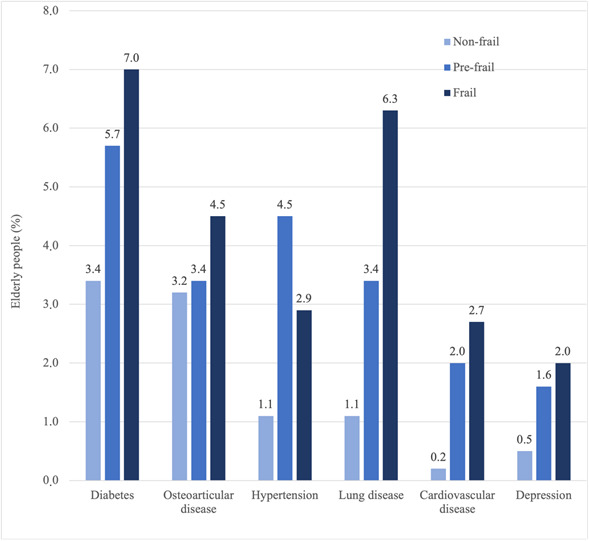
Types of therapeutic competitions per frailty category, according to the disease involved. São Paulo, 2015 (n=1,222)

The most prevalent therapeutic competitions for the general population studied involved diabetes (5.1%), osteoarticular disease (3.5%), hypertension (3.2%) and lung disease (3.0%) (Figure 1). With the exception of therapeutic competitions involving hypertension (more prevalent in the pre-frail group), all had higher prevalence in the frail group. Of the therapeutic competitions identified, two-thirds were present in pre-frail subjects. No competitions were observed with gastroesophageal reflux and benign prostatic hyperplasi.

The most frequent therapeutic competition involved the use of metformin in cases of diabetes and cardiovascular disease. For each disease pair, the medications that negatively interfere with the competing disease are presented. For example, among the 3.2% of individuals who reported hypertension as a primary disease and lung disease as a competing disease, 60.4% used beta-blockers, and 56.9% used angiotensin-converting enzyme inhibitors (Table 3).

**Table 3:** Therapeutic competitions and most frequent medications (%) according to presence of frailty and type of disease. São Paulo, 2015 (n=1,224)

Primary disease	Competing disease	Total	Frail people	Competing class or medication	Total	Frail people
Diabetes	Cardiovascular disease	4.2	6.8	Biguanides	59.0	63.7
				Angiotensin-converting enzyme inhibitors	51.7	50.3
				Metoprolol	2.4	1.7
Hypertension	Lung disease	3.2	2.9	Beta blockers	60.4	59.3
				Angiotensin-converting enzyme inhibitors	56.9	56.1
Osteoarticular disease	Hypertension	2.8	3.8	Non-steroid anti-inflammatory agents	100.0	100.0
Lung disease	Hypertension	2.4	4.7	Beta-2 agonists	94.5	94.1
				Oral corticosteroids	16.3	17.3
Cardiovascular disease	Lung disease	1.5	2.7	Beta blockers	98.1	98.0
				Non-steroid anti-inflammatory agents	8.8	5.0
				Propafenone	6.0	6.2
				Amiodarone	11.5	11.9
Osteoarticular disease	Diabetes	1.2	2.5	Non-steroid anti-inflammatory agents	100.0	100.0
Diabetes	Lung disease	1.1	1.1	Biguanides	94.9	94.9
				Metoprolol	11.9	11.9
Osteoarticular disease	Cardiovascular disease	1.0	0.5	Non-steroid anti-inflammatory agents	100.0	100.0
Lung disease	Diabetes	1.0	2.0	Beta-2 agonists	78.1	75.9
				Oral corticosteroids	29.6	32.6
Lung disease	Osteoporosis	0.9	0.9	Inhaled corticosteroids	86.0	80.4
				Oral corticosteroids	28.4	19.6

The existence of 2 therapeutic competitions in individuals with multimorbidity increased the likelihood of pre-frailty (OR 2.51; 95%CI 1.10; 5.76) (Table 4). The Hosmer-Lemeshow test for assessing quality in multinomial logistic regression had a chi-square value of 17.93 (p-value 0.328), assuming the hypothesis that the model presented a good final fit.

**Table 4:** Frailty odds ratios (OR) and 95% confidence intervals (95%CI) according to the variables of the study with older adults with multimorbidity. São Paulo, 2015

Variable	Pre-frail	Frail
Number of therapeutic competitions		
Zero	1.00	1.00
One	1.35 (0.69; 2.64)	1.40 (0.52; 3.77)
Two	2.51 (1.10; 5.76)	1.30 (0.46; 3.66)
Three or more	1.96 (0.57; 6.68)	1.86 (0.31; 11.35)
Sex		
Female	1.00	1.00
Male	1.15 (0.76; 1.73)	0.75 (0.39; 1.44)
Age group		
60-69	1.00	1.00
70-79	2.14 (1.36; 3.36)	2.77 (1.26; 6.07)
≥80	8.13 (3.50; 18.91)	39.06 (13.82; 110.40)
Schooling (years)		
8 years or more	1.00	1.00
4-7 years	1.01 (0.64; 1.60)	0.69 (0.31; 1.52)
1-3 years	1.21 (0.73; 2.00)	0.79 (0.30; 2.06)
None	1.60 (0.83; 3.09)	1.50 (0.55; 4.09)
Report of at least one fall in the last 12 months		
No	1.00	1.00
Yes	1.31 (0.92; 1.87)	1.68 (1.14; 2.48)
Hospitalization for at least one day in the last 12 months		
No	1.00	1.00
Yes	1.94 (1.06; 3.53)	4.02 (1.95; 8.28)
Self-perceived health		
Very good or good	1.00	1.00
Regular	1.49 (1.01; 2.21)	2.08 (1.12; 3.90)
Poor or very poor	2.65 (1.06; 6.63)	10.82 (3.43; 34.11)

## Discussion

This study demonstrated that 13.2% of older adults in the city of São Paulo used at least one medication capable of negatively affecting another existing disease. In frail individuals, this percentage rose by more than 5.0%. Studies that determine prevalence of therapeutic competitions in older adults are scarce, specifically those that address the prevalence of these interactions according to frailty phenotype. 

Analysis of a sample of 5,815 North American people aged 65 or over identified that 22.6% of them had therapeutic competitions ^7^. Although this prevalence rate is higher than the result found by the SABE Study, it is closer to that estimated for frail individuals (18.7%), possibly due to the greater similarity of this group to the North American sample (such as the proportion of individuals aged 80 or over and the presence of multimorbidity). 

A higher number of therapeutic competitions was identified in people who self-reported a poorer state of health and who reported a history of hospitalization in the 12 months prior to the interview. Both these characteristics were more present in frail people. Competition prevalence was also higher proportionately to the number of medications in use, this being a factor that favors the likelihood of harmful combinations in the face of other diseases. In a study with the SABE Study 2015 cohort, it was found that the likelihood of identifying therapeutic competition in individuals with multimorbidity was higher among those on polypharmacy (OR 4.70; 95%CI 3.00; 7.36), those who self-rated their health as regular, poor or very poor (OR 1.92; 95%CI 1.23; 2.99) and those with a history of hospitalization in the last 12 months (OR 1.75; 95%CI 1.07; 2.87) ^8^.

Classes of medications used in the community (such as angiotensin-converting enzyme inhibitors, biguanides and non-steroid anti-inflammatory agents) and significantly prevalent diseases (such as hypertension, diabetes and osteoarticular diseases) were involved in the most identified therapeutic competitions. This reinforces the ease with which medications used for a chronic condition can negatively affect another clinical condition in elderly people. The presence of therapeutic competitions was higher in pre-frail and frail individuals, especially in groups that had these illnesses or used these medications, when compared to non-frail groups. This result suggests that the presence of the components that define frailty syndrome, in addition to resulting in less resistance to stressors and greater vulnerability, may also be associated with other characteristics with the potential to harm health.

The high number of therapeutic competitions in pre-frail individuals stands out. It is known that pre-frailty brings with it the risk of subsequent frailty ^21^. The higher prevalence of competitions in this group may act as a contributing factor to the occurrence of frailty syndrome later, although research is still needed to prove the influence of therapeutic competitions on the frailty process.

The likelihood of pre-frailty in individuals with multimorbidity was greater in those who had two therapeutic competitions (OR 2.51; 95%CI 1.10; 5.76). This result brought relevant information about factors associated with frailty phenotype and, as a consequence, inferences about the frailty process, as the increased risk of pre-frail individuals becoming frail over time has already been demonstrated ^21^. We found that pre-frail individuals had several morbidities (62.2% of people with multimorbidity) and used multiple medications (42.2% used polypharmacy), these being profiles that increased the probability of combinations that result in therapeutic competitions.

The most prevalent therapeutic competitions were those involving diabetes, both in the general population and in groups stratified according to frailty components. The most observed therapeutic competitions were those in the presence of diabetes (primary disease) and cardiovascular diseases (competing disease), being 2.6% higher in frail individuals, when compared to the total study population. Biguanides were the medication class most involved in this competition. 

Metformin is widely used to control diabetes. Even though it has a good safety profile, use of metformin can cause adverse reactions, such as lactic acidosis, which, although rare, can be serious and potentially fatal ^22^. Due to the risk of this adverse reaction, its use may be contraindicated in chronic hypoxemic conditions, including some cardiovascular diseases ^23^. In frail people, occurrence of this reaction may be favored by changes in biological systems and decreased physiological functions, which is relevant due to the significant prevalence of this competition in this group.

Therapeutic competitions involving beta blockers with lung disease (competing disease) in the presence of hypertension or cardiovascular disease (primary disease) have also shown significant prevalence ^7^ ^,^ ^8^. Beta-blockers increased the likelihood of developing dyspnea (OR 1.10; 95%CI 1.01; 1.20) after 90 days, as demonstrated in a retrospective cohort of 10,992 post-infarction and institutionalized people aged 65 or over in the United States ^24^. This outcome occurred because some drugs in this class (such as carvedilol or propranolol) can induce bronchoconstriction in susceptible individuals ^25^.

Among frail individuals, the second most prevalent therapeutic competition was between lung disease (primary disease) and hypertension (competing disease), mainly due to the use of beta-2-agonists, but also present with oral corticosteroids. Use of beta-2-agonists can make it difficult to control blood pressure by inducing its elevation by stimulating cardiac adrenergic receptors, as can corticosteroids, which stimulate sodium reabsorption by the kidneys and enhance its vasoconstriction effect ^26^ ^,^ ^27^.

Therapeutic competition stands out with regard to the use of non-steroid anti-inflammatory agents for treatment of osteoarticular disease (primary disease), mainly with hypertension and diabetes as competing diseases. There is an important relationship between frailty and pain: persistent pain is a stressor and affects homeostasis, acting as a potentiator or inducer of frailty syndrome ^28^. Inhibition of prostaglandin synthesis by non-steroid anti-inflammatory agents interferes with control of hypertension, as it reduces its vasodilatory effect, favors sodium retention and increases aldosterone levels ^29^. In individuals with diabetes, these drugs hinder glycemic control and may expose them to a higher risk of complications induced by their nephrotoxic effect ^30^.

As far as we know, this is the first study that estimated the presence of therapeutic competitions and their main types according to frailty phenotype. Some limitations need to be highlighted. Our assessment of therapeutic competitions was restricted to the diseases listed, which could underestimate the result due to the occurrence of other unidentified diseases. Use of medications may also have been underestimated, especially those for acute conditions, which may not have been reported in the interviews. Identification of therapeutic competition did not confirm its occurrence, as its clinical consequences were not measured. 

The fact that this research was conducted using data from the SABE Study should be considered a strength. The sample used derives from population-based results, which reflect the reality of the population aged 60 and over in the São Paulo city. Although the city does not have the highest proportion of older adults in Brazil, its composition includes immigrants from different locations, giving it a mixed population that represents several regional particularities found together in the largest city in Brazil.

The results of this study highlight the need for careful assessment of the use of medications in this age group, taking into consideration and intervening with regard to characteristics of drug treatment that may negatively affect the health of this group ^31^. The participants were not exempt from the additional risks posed by pharmacotherapy, even when used for situations that require interventions. The therapeutic competitions identified, their higher prevalence in frail and pre-frail individuals and their association with pre-frailty were results that should be put to use in clinical practice. The complexity of this age group was taken into consideration, which, in general, has multiple diseases and is often on polypharmacy, and may be susceptible to negative consequences caused by the presence of therapeutic competitions. Even if a treatment is effective for a certain disease, it can compromise the individual’s safety by negatively affecting another disease that is also present.

This research found significant prevalence of therapeutic competitions in older adults. When compared to the non-frail and pre-frail groups, this result was higher among frail people. The most prevalent competitions were related to medications commonly used by the elderly population for illnesses. Increased likelihood of pre-frailty was observed in individuals with multimorbidity who had two therapeutic competitions, after adjustment for other variables.

## Data Availability

The data used in this study are available in a database that contains information about participants in the Health, Wellbeing and Aging Study (Estudo Saúde, Bem-Estar e Envelhecimento), in relation to its 2015 cohort.
