# Supplementary material for: Therapeutic competitions and frailty among older adults in São Paulo city: a cross-sectional population-based study, 2015
Source: Epidemiol Serv Saude. 2025 Apr 11;34:e20240104. doi: 10.1590/S2237-96222025v34e20240104.en (PMC11998657; doi:10.1590/S2237-96222025v34e20240104.en)
Supplement: Translation

# Translation: Competições terapêuticas e fragilidade em pessoas idosas no município de São Paulo: estudo transversal de base populacional, 2015

**Authors:** Igor Gonçalves de Souza, Yeda Aparecida de Oliveira Duarte, Mariana Martins Gonzaga do Nascimento, Caroline de Godoi Rezende Costa Molino, Cristiane de Paula Rezende, Jair Lício Ferreira Santos

**Affiliations:** 1 Universidade de São Paulo, Faculdade de Saúde Pública, São Paulo, SP, Brazil; 2 Universidade Federal de Minas Gerais, Faculdade de Farmácia, Belo Horizonte, MG, Brazil; 3 University of Zurich, Centre on Aging and Mobility, Zurich, Switzerland; 4 Universidade de São Paulo, Faculdade de Medicina de Ribeirão Preto, Ribeirão Preto, SP, Brazil

**Keywords:** Idoso, Fragilidade, Efeitos Colaterais e Reações Adversas Relacionados a Medicamentos, Interações Medicamentosas, Estudos Transversais.

## Abstract

**Objetivo:**

Identificar a presença de competições terapêuticas conforme a classificação dos componentes de fragilidade e avaliar a associação entre competições terapêuticas e fragilidade em pessoas com 60 anos ou mais no município de São Paulo.

**Métodos:**

Trata-se de estudo transversal de base populacional. Identificaram-se fragilidade, quando presentes de três a cinco componentes definidos por Fried, e pré-fragilidade, quando um ou dois estiveram presentes. Competições terapêuticas foram caracterizadas pelo uso de medicamento para determinada doença crônica não transmissível que afeta negativamente outra doença. Empregou-se regressão logística multinomial.

**Resultados:**

Identificaram-se competições terapêuticas para 13,2% dos 1.224 participantes. Desta quantidade, 18,7% foram considerados indivíduos frágeis. A competição terapêutica mais prevalente envolvia diabetes e doença cardiovascular (4,2% do total e 6,8% das pessoas frágeis) com metformina ou inibidores da enzima conversora de angiotensina. A presença de duas competições terapêuticas em indivíduos com multimorbidade aumentou a chance de pré-fragilidade (Odds ratio 2,51; intervalo de confiança 95% 1,10; 5,76).

**Conclusão:**

Competições terapêuticas são comuns na população idosa e mais frequentes entre pessoas idosas frágeis, com chance aumentada de pré-fragilidade quando duas competições terapêuticas estiveram presentes.

**Aspectos éticos**

A presente pesquisa respeitou os princípios éticos, obtendo os seguintes dados de aprovação:

Comitê de ética em pesquisa: Faculdade de Saúde Pública da Universidade de São Paulo

Número do parecer: 3.600.782

Perspectivas: 26/9/2019

Certificado de apresentação de apreciação ética: 47683115.4.0000.5421

Termo de consentimento: Obtido de todos os participantes antes da coleta

## Introdução

O crescimento da fração de pessoas idosas na população, além do desenvolvimento de tecnologias em saúde e da elevação da expectativa de vida, reflete no aumento das doenças crônicas não transmissíveis e na crescente utilização de medicamentos ^1^. Essas modificações são acompanhadas pela intensificação da prevalência de fragilidade. Tal condição é definida como síndrome multifatorial causada pela redução progressiva das reservas e resistência a agentes estressores, o que induz o declínio funcional e resulta em incapacidades, dependência e maior vulnerabilidade ^2^ ^-^ ^4^. Suas consequências incluem riscos aumentados à saúde desses indivíduos, comprometendo a independência e a autonomia, além da elevação dos custos em saúde e demanda pelo uso de mais medicamentos ^4^ ^-^ ^6^.

Ainda que necessário em diversas situações, eventualmente o medicamento aplicado para uma condição pode afetar, de forma negativa, outra doença também presente à qual não foi diretamente empregado, caracterizando a competição terapêutica. Esta é caracterizada por três componentes: i) a doença-índice, à qual ii) o medicamento que compete é empregado, e iii) a doença que compete, que é afetada de forma negativa pelo medicamento em uso ^7^. A ocorrência da competição terapêutica pode ser facilitada pela alta prevalência de indivíduos com duas ou mais doenças (multimorbidade) e pelo consequente uso de medicamentos para essas condições. Assim, ocorre a influência do medicamento utilizado para determinada doença de forma adversa a outra condição coexistente. 

A prevalência de competição terapêutica associou-se à polifarmácia, assim como à pior autoavaliação de saúde, e à ocorrência de hospitalização e queda nos 12 meses anteriores ao relato ^8^. Alguns desses fatores, como queda e hospitalização, também são conhecidos por aumentarem a chance da síndrome de fragilidade e, inclusive, de favorecer o processo de fragilização ^9^ ^,^ ^10^. Considerando a necessidade de ampliar a pesquisa no campo das competições terapêuticas na população idosa e a síndrome de fragilidade, torna-se relevante para a prática clínica definir os fatores que se associam com esse fenótipo. Esta pesquisa teve como objetivo identificar a prevalência de competições terapêuticas conforme a classificação dos componentes da fragilidade e avaliar a associação entre fragilidade e competições terapêuticas em pessoas com 60 anos ou mais residentes no município de São Paulo.

## Métodos

### Delineamento do estudo e participantes 

Trata-se de estudo transversal, de base populacional e analítico, realizado por meio dos dados provenientes do Estudo Saúde, Bem-Estar e Envelhecimento. A população do estudo foi constituída por pessoas com 60 anos ou mais residentes no município de São Paulo, de ambos os sexos, que participaram da entrevista conduzida pelo Estudo em 2015.

### Contexto

O Estudo SABE foi originado como inquérito multicêntrico que, no Brasil, foi conduzido no município de São Paulo e continuado com múltiplas coortes. Acompanha longitudinalmente os indivíduos participantes, com reaplicação do questionário estruturado em intervalos regulares. Tem como objetivo avaliar o estado de saúde e as condições de vida da população idosa, para auxiliar em saúde pública e na projeção das necessidades de pessoas dessa faixa etária ^11^.

Em 2000, foram entrevistados 2.143 indivíduos, compondo a amostra probabilística que constituiu a coorte A00. Estes foram localizados a cada reaplicação do questionário (2006, 2010 e 2015), com o acréscimo de nova coorte a cada período com indivíduos de 60 a 64 anos. Em 2015, as coortes resultaram em 1.224 indivíduos entrevistados. Os participantes foram selecionados considerando-se estratificações por sexo e grupos etários. A diferença nos números de indivíduos a cada reaplicação ocorreu em consequência de óbitos, não localização, recusas, institucionalizações e mudanças para outros municípios ^11^. A descrição completa do processo de definição da amostra e demais características metodológicas do Estudo SABE podem ser consultadas em trabalho de Lebrão et al. (2018) ^11^.

### Variável dependente: fragilidade

A variável dependente do estudo foi constituída pelo fenótipo de fragilidade. Sua definição baseou-se no conceito que determina a presença do fenótipo por cinco componentes ^3^. No Estudo SABE, estes foram definidos conforme a seguir ^9^ ^,^ ^11^.

Perda de peso não intencional - definida pela questão: “No último ano, o(a) Sr(a) perdeu peso sem fazer nenhuma dieta?”. Pontuado se perda de peso maior que 3 quilogramas.

Exaustão ou fadiga autorreferida - caracterizada por duas perguntas previamente definidas pelo Center for Epidemiological Studies - Depression e validada para indivíduos brasileiros ^12^: “Com que frequência, na última semana, a realização de suas atividades rotineiras exigiu do(a) Sr(a) um grande esforço para serem realizadas?” e “Com que frequência, na última semana, o(a) Sr.(a.) sentiu que não conseguiria levar adiante suas coisas?”. Classificavam-se os seguintes resultados: 0=nunca ou raramente (menor que um dia); 1=poucas vezes (entre um e dois dias); 2=algumas vezes (entre três e quatro dias); 3=a maior parte do tempo. Pontuado se a resposta fosse “2” ou “3” em, ao menos, um dos questionamentos.

Diminuição da força - determinada pela força de preensão manual, obtida por dinamômetro. Foram considerados, para este componente, indivíduos que constituíram o quintil mais baixo de distribuição, estratificados pelo quartil do índice de massa corporal e sexo.

Redução da velocidade de marcha - caracterizada pelo teste de caminhada de 3 metros, definido pelo Short Physical Performance Battery Assessing Lower Extremity Function ^13^. Considerado presente naqueles que constituíram o maior quintil da distribuição, segmentados pelo valor mediano da altura e sexo.

Baixo nível de atividade física - verificado pelo Physical Activity Questionnaire ^14^, em versão traduzida, pelo autorrelato da realização de atividades físicas vigorosas, moderadas ou caminhada, com posterior determinação do gasto calórico semanal pela realização das atividades relatadas. Pontuaram-se aqueles que constituíram o quintil mais baixo de gasto calórico, estratificados pelo sexo. 

Foram classificados como “frágil” aqueles indivíduos que apresentaram de três a cinco componentes do fenótipo de fragilidade. Os participantes com um ou dois componentes compuseram o grupo “pré-frágil”. Os participantes foram classificados como “não frágil” quando nenhum dos componentes estiveram presentes.

### Variáveis independentes

As variáveis independentes incluíram aquelas que representam características sociodemográficas, clínicas e comportamentais, conforme a seguir ^9^ ^,^ ^15^ ^,^ ^16^.

Faixa etária (em anos): 60-69, 70-79, 80 ou mais.

Sexo: masculino, feminino.

Escolaridade (em anos): nenhuma, 1-3, 4-7, 8 ou mais de estudo.

Arranjo domiciliar: mora acompanhado, sozinho.

Autopercepção de suficiência de renda: suficiente, insuficiente.

Tabagismo: tabagista, ex-tabagista, nunca fumou.

Consumo semanal de bebida alcoólica no último trimestre (vezes): menor que 1, 1-3, 4 ou mais.

Condições de saúde: sim, não para as seguintes doenças crônicas não transmissíveis, obtidas por perguntas específicas para cada doença no questionário do estudo - diabetes, hipertensão, depressão, doença osteoarticular, osteoporose, doença cardiovascular, doença pulmonar, doença de Alzheimer, hipercolesterolemia, hiperplasia prostática benigna e doença do refluxo gastroesofágico.

Medicamentos em uso: codificados pela classificação Anatomical Therapeutic Chemical. Polifarmácia foi definida como uso de cinco medicamentos ou mais.

Hospitalização por pelo menos um dia nos últimos 12 meses: sim, não.

Relato de pelo menos uma queda nos últimos 12 meses: sim, não.

Autopercepção de saúde: boa/muito boa, regular, muito ruim/ruim.

Empregou-se como variável independente a presença de competições terapêuticas, previamente elencadas por Molino (2018) pela seleção de guias de prática clínica de alta qualidade elencados por avaliação da qualidade e revisão sistemática. Esses documentos apresentam o conhecimento científico sobre determinado assunto de modo sintetizado, fornecendo recomendações práticas para os profissionais da saúde ^8^ ^,^ ^17^ ^,^ ^18^. Foram empregados guias de prática clínica nacionais e internacionais de alta qualidade para identificação de competições terapêuticas em pessoas idosas com enfermidades ^8^. Neste estudo, verificou-se a competição terapêutica no grupo de participantes com multimorbidade, presente nos indivíduos com pelo menos duas das doenças elencadas anteriormente e de maior prevalência em pessoas idosas na comunidade ^19^.

Considerou-se competição terapêutica quando o participante relatou utilizar o medicamento que compete e apresentava determinada doença-índice na presença da doença que compete. Não foram considerados outros tipos de interações fármaco-doença neste estudo. Combinações em dose fixa também foram utilizadas na identificação das competições quando pelo menos um dos fármacos era causador da competição. 

### Métodos estatísticos

As frequências relativas das variáveis qualitativas foram apresentadas na análise descritiva dos dados, assim como as médias daquelas quantitativas, com os desvios-padrão. As análises comparativas das variáveis categóricas competição terapêutica e fragilidade foram realizadas empregando-se o teste qui-quadrado de Pearson com correção Rao-Scott. Consideraram-se o nível de significância de 5% e os pesos amostrais para estimativas com ponderações populacionais.

As análises univariada e múltipla foram realizadas para o grupo de indivíduos com multimorbidade, já que este é o grupo predisposto para a presença de competições terapêuticas. Foram selecionadas variáveis com nível de significância menor ou igual a 0,20 na análise univariada para inclusão no modelo múltiplo. Este foi realizado por regressão logística multinomial, sendo o fenótipo de fragilidade a variável dependente, constituída pelos grupos “não frágil”, “pré-frágil” e “frágil”. O número de competições terapêuticas foi incluído no modelo múltiplo como variável independente, categorizada em “nenhuma”, “uma”, “duas” e “três ou mais competições terapêuticas”, sendo a categoria “nenhuma” utilizada como referência. O valor de significância estatística de 5% foi adotado. Os resultados foram apresentados pela razão de chances (odds ratio, OR) e intervalos de confiança de 95% (IC95%). O teste de qualidade de Hosmer-Lemeshow para regressão logística multinomial foi utilizado para avaliação da qualidade do ajuste do modelo múltiplo final, admitindo-se a hipótese nula de que o ajuste do modelo está adequado ^20^. 

Utilizou-se o software Stata (versão 14) para a realização das análises estatísticas. Os pesos amostrais foram considerados nas análises e, portanto, os resultados apresentados podem ser extrapolados para a população com 60 anos ou mais no município de São Paulo.

## Resultados

Foram incluídos 1.124 indivíduos. A média de idade foi 70,8±0,7 anos, e 56,7% eram do sexo feminino. Possuíam pelo menos 1 morbidade 85,6% dos participantes, sendo hipertensão (66,3%), doença osteoarticular (33,5%) e diabetes (28,2%) as mais prevalentes. Dos entrevistados, 28,7% reportaram 2 doenças e 33,2% possuíam 3 ou mais, tendo em média 2,0±0,1 enfermidades. 9 em cada 10 indivíduos relataram utilizar pelo menos 1 medicamento (85,4%). Mais de um terço fazia uso de polifarmácia (38,8%), e 6,4%, polifarmácia excessiva (10 ou mais medicamentos em uso). Os participantes usavam, em média, 4,1±0,2 medicamentos.

Foram identificados como frágeis 11,2% dos indivíduos, 56,1% pré-frágeis e 32,7% não frágeis. A maioria dos participantes que não utilizavam medicamento pertencia ao grupo de pré-frágeis ou não frágeis (97,0%). O uso de pelo menos 1 medicamento foi feito por 96,2% dos indivíduos frágeis e 87,7% dos pré-frágeis. Este grupo apresentou polifarmácia por 42,2% dos indivíduos. Sexo feminino, menor nível de escolaridade e o grupo de 80 anos ou mais foram os que apresentaram maior prevalência de indivíduos frágeis (Tabela 1). Multimorbidade foi identificada em 78,9% das pessoas frágeis, 62,2% nos pré-frágeis e 60,7% nos não frágeis.

**Tabela 1:** Características (%) das pessoas idosas pelo total de entrevistados, conforme fenótipo de fragilidade, São Paulo, 2015 (n=1.224)

Variáveis	Total	Não frágil	Pré-frágil	Frágil	p-valor
Sexo					0,027
Feminino	56,7	33,5	53,2	13,3	
Masculino	43,3	31,7	59,7	8,6	
Faixa etária (anos)					<0,001
60-69	54,3	43,8	50,9	5,3	
70-79	30,5	25,9	64,3	9,8	
≥80	15,2	7,0	58,0	35,0	
Escolaridade (anos)					<0,001
Nenhuma	13,1	18,9	60,3	20,8	
1-3	17,9	28,4	57,6	14,0	
4-7	37,3	34,6	55,6	9,8	
≥8	31,7	38,9	54,2	6,9	
Arranjo domiciliar					0,466
Mora acompanhado	84,3	32,4	56,7	10,9	
Mora sozinho	15,7	34,9	52,3	12,8	
Percepção de renda					0,813
Suficiente	52,7	32,8	56,7	10,5	
Insuficiente	47,3	32,8	55,5	11,7	
Plano de saúde					0,205
Não	53,6	31,0	56,5	12,5	
Sim	46,4	34,8	55,5	9,7	
Relato de pelo menos uma queda nos últimos 12 meses					0,058
Não	71,2	34,5	56,1	9,4	
Sim	28,8	28,6	55,8	15,6	
Hospitalização por pelo menos um dia nos últimos 12 meses (n=1.220)					<0,001
Não	86,8	35,1	55,8	9,1	
Sim	13,2	17,5	57,2	25,3	
Tabagismo (n=1.218)					0,046
Nunca fumou	48,3	34,2	52,5	13,3	
Ex-tabagista	38,4	30,2	60,9	8,9	
Tabagista	48,3	35,4	55,3	9,3	
Consumo semanal de bebida alcoólica no trimestre (vezes) (n=1.218)					0,056
<1	81,1	32,4	55,1	12,5	
1-3	11,7	37,8	56,9	5,3	
4 ou mais	7,2	28,3	65,4	6,3	
Autopercepção de saúde (n=1.189)					<0,001
Muito boa ou boa	49,2	39,7	53,7	6,6	
Regular	44,1	29,4	60,1	10,5	
Ruim ou muito ruim	6,7	13,0	56,5	30,5	
Uso de medicamentos					<0,001
Nenhum	14,6	50,0	47,0	3,0	
1-2	21,9	35,4	53,8	10,8	
3-4	24,7	31,4	55,7	12,9	
5 ou mais	38,8	25,6	60,9	13,5	
Multimorbidade (n=1.222)					0,001
Não	36,5	37,1	56,5	6,4	
Sim	63,5	30,0	55,7	14,3	
Hipertensão (n=1.221)					0,009
Não	33,7	37,5	54,9	7,6	
Sim	66,3	30,4	56,7	12,9	
Diabetes (n=1.221)					0,029
Não	71,8	32,2	58,1	9,7	
Sim	28,2	33,6	51,5	14,9	
Doença pulmonar (n=1.221)					0,009
Não	91,8	22,5	62,5	15,0	
Sim	8,2	33,7	55,6	10,7	
Doença cardiovascular (n=1.221)					<0,001
Não	76,2	36,0	54,1	9,9	
Sim	23,8	22,1	62,5	15,4	
Doença osteoarticular (n=1.221)					0,049
Não	66,5	33,7	56,6	9,7	
Sim	33,5	31,5	54,3	14,2	
Depressão (n=1.220)					0,025
Não	84,0	33,4	56,4	10,2	
Sim	16,0	29,6	54,0	16,4	
Osteoporose (n=1.221)					0,012
Não	73,8	33,7	56,3	10,0	
Sim	26,2	27,9	54,9	17,2	
Doença de Alzheimer (n=1.220)					<0,001
Não	97,9	33,4	56,3	10,3	
Sim	2,1	3,7	45,8	50,5	
Doença do refluxo gastroesofágico (n=1.222)					0,840
Não	98,1	32,7	56,1	11,2	
Sim	1,9	34,2	50,8	15,0	
Hiperplasia prostática benigna (n=1.222)					0,645
Não	99,8	32,7	56,1	11,2	
Sim	0,25	56,6	43,4	0	
Hipercolesterolemia (n=1.222)					0,219
Não	96,6	32,3	56,3	11,4	
Sim	3,4	43,2	50,7	6,1	
Competição terapêutica (n=1.222)					0,015
Não	86,8	34,5	55,0	10,5	
Sim	13,2	21,4	62,8	15,8	

A prevalência de competições terapêuticas foi 13,2% entre os participantes, o que indica que 1 a cada 10 indivíduos com 60 anos ou mais no município de São Paulo utilizava pelo menos 1 medicamento capaz de afetar negativamente outra doença presente. Considerando-se o total de participantes, 8,0% apresentaram 1 competição terapêutica, 2,8% apresentaram 2, e 2,4%, 3 ou mais. O percentual de indivíduos com competição terapêutica foi crescente conforme o fenótipo de fragilidade, demonstrando-se 10,0% superior em frágeis, em comparação àqueles não frágeis (Tabela 2).

**Tabela 2:** Características (%) das pessoas idosas conforme presença de competição terapêutica. São Paulo, 2015 (n=1.224)

Variáveis	Sem competição terapêutica	Com competição terapêutica	p-valor
**Sexo**			0,044
Feminino	84,9	15,1	
Masculino	89,2	10,8	
**Faixa etária (anos)**			0,058
60-69	89,2	10,8	
70-79	84,6	15,4	
80 ou mais	82,6	17,4	
**Escolaridade (anos)**			0,238
Nenhuma	86,6	13,4	
1-3 anos	84,2	15,8	
4-7 anos	85,7	14,3	
8 anos ou mais	89,6	10,4	
**Arranjo domiciliar**			0,932
Mora acompanhado	86,8	13,2	
Mora sozinho	86,6	13,4	
**Percepção de renda**			0,739
Suficiente	87,1	12,9	
Insuficiente	86,4	13,6	
**Plano de saúde**			0,499
Não	87,5	12,5	
Sim	86,0	14,0	
**Relato de pelo menos uma queda nos últimos 12 meses**			0,010
Não	88,7	11,3	
Sim	82,1	17,9	
**Hospitalização por pelo menos um dia nos últimos 12 meses**			<0,001
Não	88,3	11,7	
Sim	76,7	23,3	
**Tabagismo**			0,971
Nunca fumou	87,0	13,0	
Ex-tabagista	86,5	13,5	
Tabagista	86,5	13,5	
**Consumo semanal de bebida alcoólica no trimestre (vezes)**			0,398
<1	86,2	13,8	
1-3	88,4	11,6	
4 ou mais	90,9	9,1	
**Autopercepção de saúde**			<0,001
Muito boa ou boa	91,2	8,8	
Regular	84,1	15,9	
Ruim ou muito ruim	70,4	29,6	
**Uso de medicamentos**			<0,001
Nenhum	100,0	0	
1-2	96,4	3,6	
3-4	88,3	11,7	
5 ou mais	75,4	24,6	
**Multimorbidade**			<0,001
Não	100,0	0	
Sim	78,6	21,4	
**Hipertensão**			<0,001
Não	95,6	4,4	
Sim	82,3	17,7	
**Diabetes**			<0,001
Não	90,8	9,2	
Sim	76,5	23,5	
**Doença pulmonar**			<0,001
Não	91,7	8,3	
Sim	32,3	67,7	
**Doença cardiovascular**			<0,001
Não	92,4	7,6	
Sim	68,6	31,4	
**Doença osteoarticular**			<0,001
Não	90,6	9,4	
Sim	78,7	21,3	
**Depressão**			<0,001
Não	88,4	11,6	
Sim	78,3	21,7	
**Osteoporose**			<0,001
Não	88,4	11,6	
Sim	78,0	22,0	
**Doença de Alzheimer**			0,956
Não	86,8	13,2	
Sim	86,4	13,6	
**Doença do refluxo gastroesofágico**			0,695
Não	86,7	13,3	
Sim	89,7	10,3	
**Hiperplasia prostática benigna**			0,038
Não	86,9	13,1	
Sim	43,4	56,6	
**Hipercolesterolemia**			0,092
Não	87,1	12,9	
Sim	78,1	21,9	
**Fenótipo de fragilidade**			0,015
Não frágil	91,4	8,6	
Pré-frágil	85,2	14,8	
Frágil	81,3	18,7	

As competições terapêuticas mais prevalentes para a população geral do estudo envolveram diabetes (5,1%), doença osteoarticular (3,5%), hipertensão (3,2%) e doença pulmonar (3,0%) (Figura 1). Com exceção das competições terapêuticas envolvendo hipertensão (mais prevalente no grupo de pré-frágeis), todas apresentaram prevalência mais elevada no grupo de frágeis. Das competições terapêuticas identificadas, dois terços estavam presentes em pré-frágeis. Não foram observadas competições com doença do refluxo gastroesofágico e hiperplasia prostática benigna.

**Figura 1:** Tipos de competições terapêuticas por categorias de fragilidade, de acordo com a doença envolvida. São Paulo, 2015 (n=1.222)

A competição terapêutica mais frequente envolveu a utilização de metformina na presença de diabetes e doença cardiovascular. Para cada dupla de enfermidade, apresentaram-se os medicamentos que interferem negativamente na doença que compete. Por exemplo, dos 3,2% indivíduos que relataram doença-índice hipertensão e doença pulmonar como doença que compete, 60,4% usavam betabloqueadores, e 56,9%, inibidores da enzima conversora de angiotensina (Tabela 3).

**Tabela 3:** Competições terapêuticas e medicamentos mais frequentes (%) pela presença de fragilidade e tipo de doença, São Paulo, 2015 (n=1.224)

Doença índice	Doença que compete	Total	Pessoas frágeis	Classe ou medicamento que compete	Total	Pessoas frágeis
Diabetes	Doença cardiovascular	4,2	6,8	Biguanidas	59,0	63,7
				Inibidores da enzima conversora de angiotensina	51,7	50,3
				Metoprolol	2,4	1,7
Hipertensão	Doença pulmonar	3,2	2,9	Beta-bloqueadores	60,4	59,3
				Inibidores da enzima conversora de angiotensina	56,9	56,1
Doença osteoarticular	Hipertensão	2,8	3,8	Anti-inflamatórios não esteroides	100,0	100,0
Doença pulmonar	Hipertensão	2,4	4,7	Beta-2 agonistas	94,5	94,1
				Corticosteroides de uso oral	16,3	17,3
Doença cardiovascular	Doença pulmonar	1,5	2,7	Beta-bloqueadores	98,1	98,0
				Anti-inflamatórios não esteroides	8,8	5,0
				Propafenona	6,0	6,2
				Amiodarona	11,5	11,9
Doença osteoarticular	Diabetes	1,2	2,5	Anti-inflamatórios não esteroides	100,0	100,0
Diabetes	Doença pulmonar	1,1	1,1	Biguanidas	94,9	94,9
				Metoprolol	11,9	11,9
Doença osteoarticular	Doença cardiovascular	1,0	0,5	Anti-inflamatórios não esteroides	100,0	100,0
Doença pulmonar	Diabetes	1,0	2,0	Beta-2 agonistas	78,1	75,9
				Corticosteroides orais	29,6	32,6
Doença pulmonar	Osteoporose	0,9	0,9	Corticosteroides inalatórios	86,0	80,4
				Corticosteroides orais	28,4	19,6

A existência de 2 competições terapêuticas em indivíduos com multimorbidade aumentou a chance de pré-fragilidade (OR 2,51; IC95% 1,10; 5,76) (Tabela 4). O teste de Hosmer-Lemeshow para avaliação da qualidade em regressão logística multinomial teve valor de qui-quadrado de 17,93 (p-valor 0,328), admitindo-se a hipótese de que o modelo apresentou bom ajuste final.

**Tabela 4:** Razão de chances (odds ratio, OR) e intervalo de confiança de 95% (IC95%) da fragilidade pelas variáveis do estudo em pessoas idosas com multimorbidade, São Paulo, 2015 (n=775)

Variável	Pré-frágil	Frágil
Número de competições terapêuticas		
Zero	1,00	1,00
Uma	1,35 (0,69; 2,64)	1,40 (0,52; 3,77)
Duas	2,51 (1,10; 5,76)	1,30 (0,46; 3,66)
Três ou mais	1,96 (0,57; 6,68)	1,86 (0,31; 11,35)
Sexo		
Feminino	1,00	1,00
Masculino	1,15 (0,76; 1,73)	0,75 (0,39; 1,44)
Faixa etária (anos)		
60-69	1,00	1,00
70-79	2,14 (1,36; 3,36)	2,77 (1,26; 6,07)
≥80	8,13 (3,50; 18,91)	39,06 (13,82; 110,40)
Escolaridade (anos)		
8 ou mais	1,00	1,00
4-7 anos	1,01 (0,64; 1,60)	0,69 (0,31; 1,52)
1-3 anos	1,21 (0,73; 2,00)	0,79 (0,30; 2,06)
Nenhuma	1,60 (0,83; 3,09)	1,50 (0,55; 4,09)
Relato de pelo menos uma queda nos últimos 12 meses		
Não	1,00	1,00
Sim	1,31 (0,92; 1,87)	1,68 (1,14; 2,48)
Hospitalização por pelo menos um dia nos últimos 12 meses		
Não	1,00	1,00
Sim	1,94 (1,06; 3,53)	4,02 (1,95; 8,28)
Autopercepção de saúde		
Muito boa ou boa	1,00	1,00
Regular	1,49 (1,01; 2,21)	2,08 (1,12; 3,90)
Ruim ou muito ruim	2,65 (1,06; 6,63)	10,82 (3,43; 34,11)

## Discussão

Este trabalho demonstrou que 13,2% das pessoas idosas no município de São Paulo utilizavam pelo menos um medicamento capaz de afetar negativamente outra morbidade existente. Em indivíduos frágeis, esse percentual se elevou em mais de 5,0%. Trabalhos que determinam a prevalência de competições terapêuticas em pessoas idosas são escassos, especificamente aqueles que abordem a prevalência dessas interações conforme o fenótipo de fragilidade. 

Em um recorte de 5.815 pessoas norte-americanas com 65 anos ou mais, identificou-se que 22,6% destas apresentavam competições terapêuticas ^7^. Embora a prevalência seja superior ao resultado encontrado pelo Estudo SABE, essa prevalência aproxima-se mais daquele estimado em indivíduos frágeis (18,7%), possivelmente devido à maior semelhança desse grupo à amostra de norte-americanos (como a fração de indivíduos com 80 anos ou mais e a presença de multimorbidade). 

Número mais elevado de competições terapêuticas foi identificado em pessoas que autodeclararam pior estado de saúde e que relataram histórico de hospitalização nos 12 meses anteriores à entrevista, ambas as características mais presentes em pessoas frágeis. A prevalência de competições também foi mais elevada proporcionalmente ao número de medicamentos em uso, fator este que favorece a probabilidade de combinações prejudiciais diante de outras doenças. Em estudo com a coorte de 2015 do Estudo SABE, observou-se que a chance de identificar competição terapêutica em indivíduos com multimorbidade foi mais elevada naqueles em polifarmácia (OR 4,70; IC95% 3,00; 7,36), que autoavaliaram a saúde como regular, ruim ou muito ruim (OR 1,92; IC95% 1,23; 2,99) e com histórico de hospitalização nos últimos 12 meses (OR 1,75; IC95% 1,07; 2,87) ^8^.

Classes de medicamentos utilizados na comunidade (como inibidores da enzima conversora de angiotensina, biguanidas e anti-inflamatórios não esteroides) e doenças significativamente prevalentes (como hipertensão, diabetes e doenças osteoarticulares) foram envolvidas nas competições terapêuticas mais identificadas. Isso reforça a facilidade com que medicamentos de uso crônico podem afetar negativamente outra condição clínica na pessoa idosa. A presença de competições terapêuticas foi mais elevada em indivíduos pré-frágeis e frágeis, especialmente nos grupos que apresentavam essas enfermidades ou utilizavam esses medicamentos, quando comparados aos não frágeis. Esse resultado sugere que a presença dos componentes que definem a síndrome de fragilidade, além de resultarem em menor resistência a estressores e maior vulnerabilidade, podem também estar associados a outras características com potencial de trazer prejuízo à saúde. 

Ressalta-se o número elevado de competições terapêuticas em indivíduos pré-frágeis. O risco de fragilização posterior, conferido pelo estado de pré-fragilidade, é conhecido ^21^. A prevalência mais elevada de competições nesse grupo pode atuar como fator contribuinte para ocorrência da síndrome de fragilidade posteriormente, embora pesquisas ainda sejam necessárias para comprovar a influência das competições terapêuticas no processo de fragilização. 

A chance de pré-fragilidade em indivíduos com multimorbidade foi maior naqueles que apresentaram duas competições terapêuticas (OR 2,51; IC95% 1,10; 5,76). Esse resultado trouxe informações relevantes sobre fatores associados ao fenótipo de fragilidade e, como consequência, inferências sobre o processo de fragilização, pois o risco aumentado de que indivíduos pré-frágeis tornem-se frágeis ao longo do tempo já foi demonstrado ^21^. Verificou-se que indivíduos pré-frágeis tinham várias morbidades (62,2% das pessoas com multimorbidade) e utilizavam múltiplos medicamentos (42,2% faziam uso de polifarmácia), perfis que elevavam a probabilidade de combinações que resultem em competições terapêuticas. 

As competições terapêuticas mais prevalentes foram aquelas envolvendo diabetes, tanto na população geral quanto nos grupos estratificados pelos componentes de fragilidade. As competições terapêuticas mais observadas foram aquelas na presença de diabetes (doença-índice) e doenças cardiovasculares (doença que compete), 2,6% maior em indivíduos frágeis, quando comparados à população total do estudo. As biguanidas constituíram a classe mais envolvida nessa competição. 

A metformina é amplamente utilizada para controle do diabetes. Ainda que apresente bom perfil de segurança, o uso de metformina pode ocasionar reações adversas, como acidose láctica, que, embora seja rara, pode ser grave e potencialmente fatal ^22^. Devido ao risco dessa reação adversa, sua utilização pode ser contraindicada em condições hipoxêmicas crônicas, incluindo algumas doenças cardiovasculares ^23^. Em pessoas frágeis, a ocorrência dessa reação pode ser favorecida pelas alterações em sistemas biológicos e diminuição das funções fisiológicas, relevante pela prevalência expressiva dessa competição nesse grupo.

As competições terapêuticas envolvendo betabloqueadores com doença pulmonar (doença que compete) na presença de hipertensão ou doença cardiovascular (doença-índice) também apresentaram prevalência significativa ^7^ ^,^ ^8^. Betabloqueadores aumentaram a chance de desenvolver dispneia (OR 1,10; IC95% 1,01; 1,20) após 90 dias, conforme demonstrado em coorte retrospectiva com 10.992 pessoas com 65 anos ou mais em pós-infarto e institucionalizadas nos Estados Unidos ^24^. Esse desfecho ocorreu, pois alguns fármacos dessa classe (como carvedilol ou propranolol) podem induzir broncoconstrição em indivíduos susceptíveis ^25^.

Em indivíduos frágeis, a segunda competição terapêutica mais prevalente foi entre doença pulmonar (doença-índice) e hipertensão (doença que compete), principalmente pelo uso de beta-2-agonistas, mas também presentes com corticosteroides de uso oral. O uso de beta-2-agonista pode dificultar o controle da pressão arterial por induzir sua elevação ao estimular receptores adrenérgicos cardíacos, assim como os corticosteroides, que estimulam a reabsorção sódio pelos rins e potencializam seu efeito vasoconstritor ^26^ ^,^ ^27^.

Destaca-se a competição terapêutica pelo uso de anti-inflamatórios não esteroides para tratamento de doença osteoarticular (doença-índice), principalmente tendo hipertensão e diabetes como doenças que competem. Existe importante relação entre fragilidade e dor: a dor persistente é um agente estressor e afeta a homeostase, atuando como potencializador ou indutor da síndrome de fragilidade ^28^. A inibição da síntese de prostaglandinas pelos anti-inflamatórios não esteroides interfere no controle da hipertensão, pois reduz seu efeito vasodilatador, favorece a retenção de sódio e eleva os níveis de aldosterona ^29^. Em indivíduos com diabetes, esses fármacos dificultam o controle glicêmico e podem os expor ao risco mais elevado de complicações induzidas pelo seu efeito nefrotóxico ^30^.

Até onde se sabe, este é o primeiro trabalho que estimou a presença de competições terapêuticas e seus principais tipos conforme fenótipo de fragilidade. Algumas limitações devem ser pontuadas. A avaliação das competições terapêuticas foi restrita às doenças elencadas, podendo subestimar o resultado pela ocorrência de outras não identificadas. O uso de medicamentos também pode ter sido subestimado, especialmente aqueles para condições agudas, que eventualmente não foram relatados nas entrevistas. A identificação de competição terapêutica não confirmou sua ocorrência, visto que suas consequências clínicas não foram mensuradas. 

A realização desta pesquisa por meio de dados do Estudo SABE deve ser considerada ponto forte. A amostra empregada decorre de resultados de base populacional, que refletem a realidade da população com 60 anos ou mais no município. Apesar de a cidade não apresentar a maior proporção de pessoas idosas no país, sua composição inclui imigrantes de diversas localidades, conferindo uma população miscigenada e que representa várias particularidades regionais na maior cidade do Brasil.

Os resultados deste estudo ressaltaram a necessidade de avaliação criteriosa do uso de medicamentos nesse grupo etário, considerando e intervindo sobre características do tratamento medicamentoso que podem afetar a saúde desse grupo negativamente ^31^. Os indivíduos não foram isentos dos riscos adicionais predispostos pela farmacoterapia, mesmo que empregada para situações que necessitam de intervenções. As competições terapêuticas identificadas, a sua prevalência mais elevada em indivíduos frágeis e pré-frágeis e a associação com pré-fragilidade foram resultados que devem ser empregados na prática clínica. Considerou-se a complexidade desse grupo etário que, em geral, possui múltiplas doenças e frequentemente está em polifarmácia, podendo estar susceptíveis às consequências negativas ocasionadas pela presença de competições terapêuticas. Ainda que um tratamento seja efetivo para determinada doença, este pode comprometer a segurança do indivíduo por afetar negativamente outra enfermidade também presente.

Esta pesquisa constatou prevalência expressiva de competições terapêuticas em pessoas idosas. Quando comparado aos grupos de não frágeis e pré-frágeis, esse resultado foi superior em pessoas frágeis. As competições mais prevalentes estiveram relacionadas com medicamentos usualmente utilizados pela população idosa para as doenças. Observou-se o aumento da chance de pré-fragilidade em indivíduos com multimorbidade que possuíam duas competições terapêuticas, após ajuste a outras variáveis.

## Data Availability

Os dados empregados no estudo estão disponíveis em banco de dados que contém as informações dos participantes do Estudo Saúde, Bem-Estar e Envelhecimento, referente à coorte de 2015.
